# Cytokine (IL-10, IL-6, TNF-α and TGF-β1) Gene Polymorphisms in Chronic Hepatitis C Virus Infection among Malay Male Drug Abusers

**DOI:** 10.3390/biomedicines9091115

**Published:** 2021-08-30

**Authors:** Ismail Che Noh, Imran Ahmad, Siti Suraiya, Nur Fadhlina Musa, Asma Abdullah Nurul, Abu Bakar Ruzilawati

**Affiliations:** 1 Department of Pharmacology, School of Medical Sciences, Universiti Sains Malaysia, Kelantan 16150, Malaysia; ismailnoh83@gmail.com; 2 Faculty of Medicine and Health Sciences, Universiti Malaysia Sabah, Sabah 88400, Malaysia; 3 Department of Family Medicine, School of Medical Sciences, Universiti Sains Malaysia, Kelantan 16150, Malaysia; profimran@usm.my; 4 Department of Medical Microbiology and Parasitology, School of Medical Sciences, Universiti Sains Malaysia, Kelantan 16150, Malaysia; ssuraiya@usm.my; 5 Human Genome Centre, School of Medical Sciences, Universiti Sains Malaysia, Kelantan 16150, Malaysia; fadhlina@usm.my; 6 Biomedicine Programme, School of Health Sciences, Universiti Sains Malaysia, Kelantan 16150, Malaysia; nurulasma@usm.my

**Keywords:** cytokines, gene polymorphism, hepatitis C infection, drug abusers, Malay male

## Abstract

Cytokines play an important role in modulating inflammation during viral infection, including hepatitis C virus (HCV) infection. Genetic polymorphisms of cytokines can alter the immune response against this infection. The objective of this study was to investigate the possible association between chronic hepatitis C virus infection susceptibility and cytokine gene polymorphism for interleukin-10 (IL-10) rs1800896 and rs1800871, interleukin 6 (IL-6) rs1800795, TNF-α rs1800629, and TGF-β1 rs1800471 in Malay male drug abusers. The study was conducted on 76 HCV-positive (HP) male drug abusers and 40 controls (HCV-negative male drug abusers). We found that there were significant differences in the frequencies of genotype for IL-10 rs1800871 (*p* = 0.0386) and at the allelic level for IL-10 rs1800896 A versus G allele (*p* = 0.0142) between the HP group and the control group. However, there were no significant differences in gene polymorphism in interleukin 6 rs1800795, TNF-α rs1800629 and TGF-β1 rs1800471. These findings suggest significant associations between gene polymorphism for IL-10 rs1800871, IL-10 rs1800896 (at the allelic level) and susceptibility to HCV infection among Malay male drug abusers.

## 1. Introduction

Hepatitis C virus (HCV) infection is the leading cause of chronic liver disease and liver cancer. It is estimated that the worldwide prevalence of HCV was around 1% in 2015, accounting for 71.1 million people burdened by the disease [1]. HCV is a bloodborne virus that is transmitted frequently via the usage of intravenous drugs, contributing to 60–80% of HCV cases in developed countries [2]. The risk of infection is significantly increased for those with a history of injection of 6 years and more [3]. Other risk factors for the spread include unscreened blood products for transfusion, unsafe sexual practices, and vertical transmission [4].

About 15–45% of patients infected with HCV spontaneously recover from the disease without any treatment, while the majority of patients develop persistent chronic HCV [5]. Between 5 and 20% of chronic HCV patients develop liver cirrhosis, with a significant risk of developing complications, such as liver cancer, liver failure, and increase mortality [6]. This could signify host genetic differences—in the response and the course of disease. HCV itself is not cytopathic. The lesion of chronic hepatitis C appears to be due to a local immune response, with cytokines playing a major role in modulating this response [7].

Cytokines are glycoproteins or humoral immunomodulatory proteins secreted by a wide range of cells, including immune cells, such as macrophages, T or B lymphocytes, and mast cells, as well as endothelial cells, fibroblasts, and stromal cells [8]. Cytokines can be broadly classified as monokines (produced by monocytes’ lineage) or lymphokines (produced by lymphocytes lineage). They can also be classified based on function: type 1 are the pro-inflammatory cytokines (e.g., tumor necrosis factor (TNF)-α, interferon-γ, interleukin-2, and interleukin-12), and type 2 are the anti-inflammatory cytokines (e.g., tumor growth factor (TGF)-β1, interleukin-4, interleukin-5, interleukin-6, interleukin-10, and interleukin-13) [9]. Type 1 enhances the cellular immune response, and type 2 favors the antibody response. However, certain cytokines, such as Il-6, demonstrate both pro- and anti-inflammatory effects.

The synthesis and release of cytokines from innate immune cells is a fundamental response to inflammation and infection in the body. The dysregulation of pro-inflammatory and anti-inflammatory cytokine profiles may be involved in the pathogenesis and influence the clinical outcome of many infectious, autoimmune, and malignant diseases [10]. The imbalance between the interactions of these two types of cytokines leads to the disruption of the normal feedback mechanism and threatens normal tissue integrity. The overexpression of type 1 cytokines can lead to widespread inflammatory reactions and significant adverse events.

There are numerous studies on the relationship between the genetic polymorphism within a particular gene (which may influence the level of expression of cytokines) and individual variations in clinical features, such as susceptibility to infection or the progression of certain diseases. The differences in cytokine profiles among individuals can be due to allelic polymorphism that occurs within regulatory or coding regions of cytokines gene. Non-conservative mutation within the coding region can result in loss, abrogation, or change of function in the expressed protein, whereas polymorphism within the 5′- and 3′- regulatory sequences or introns may affect transcription [11]. This study’s main objective was to investigate the possible association between chronic hepatitis C virus infection susceptibility and cytokine gene polymorphism for interleukin-10 (IL-10) (rs1800896 and rs1800871), interleukin 6 (IL-6) rs1800795, TNF-α rs1800629, and TGF-β1 rs1800471 in Malay male drug abusers.

## 2. Materials and Methods

### 2.1. Subjects and Ethical Clearance

This study received ethical clearances from the Medical Research & Ethics Committee, Ministry of Health Malaysia (NMRR-19-399-45866) and USM Human Research Ethics Committee (USM/JEPeM/18010012). Subjects’ recruitment was conducted in various health clinics in the state of Kelantan, Malaysia, between July 2019 and December 2020. A total of 116 adult male subjects (76 drug abusers with chronic HCV and 40 drug abusers without HCV as controls) were enrolled in this study according to the inclusion and exclusion criteria. The inclusion criteria for the drug abuser group included adults (≥18 years old) with a history of drug dependence based on an assessment of the structured clinical review DSM-V. The exclusion criteria were patients with liver disease of etiology other than HCV or mental illness who refused or were unable to give informed consent. All participants were subjected to a medical history and physical examination. Diagnosis of chronic HCV infection was based on the persistence of positive HCV RNA for at least 6 months.

### 2.2. DNA Extraction

A total of 10 mL of the blood sample was collected into a sterile tube containing heparin K2EDTA blood collection tubes. The genomic DNA was extracted using a QIAamp DNA blood mini kit (Qiagen, Hilden, Germany) with lot no 51104 according to manufacturer instructions. Spectrophotometry was used to determine the purity and concentration of DNA extracts. The quality of the DNA was reflected by a consistent ratio of 1.8 to 2.0. Then, the coded genomic DNA solution was stored at 4 °C.

### 2.3. Genetic Study

The single nucleotide mutations of interleukin 10 rs1800896, rs1800871, interleukin 6 rs1800795, TNF-α rs1800629 and TGF-β1 rs1800471 were analyzed using the multiplex polymerase chain reaction (PCR) method. Two separate multiplex PCR were performed based on a suitable combination of primers and annealing temperature. IL-10 primers rs1800896 and rs1800871 were combined with IL-6 rs1800795 primer whereas TNF-α primer rs1800629 was combined with TGF-β1 rs1800471 primer. Each PCR setup consisted of two reactions, with each reaction containing a combination of one of the two alleles specific for forward and reverse primers (Table 1). For IL-10 and IL-6, the first reaction (wild type) was a combination of IL-10 Primer A and T with IL-6 Primer G, while the second reaction (mutant type) was a combination of IL-10 Primer G and C with IL-6 Primer C. Whereas for TNF-α and TGF-β1, the first reaction (wild type) was a combination of TNF-α Primer G with TGF-β1 Primer G and the second reaction (mutant type) was a combination of TNF-α Primer A with TGF-β1 Primer C.

**Figure 1:**
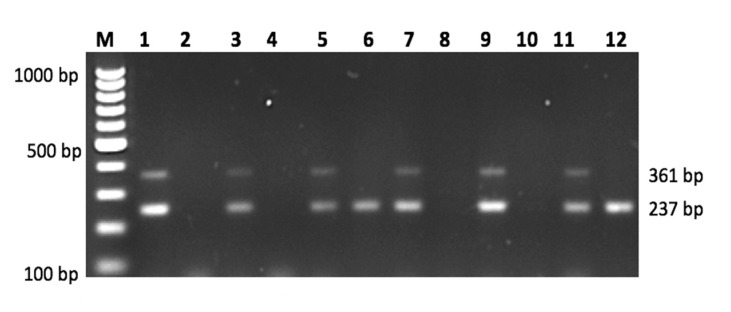
Multiplex PCR consist of IL-10 rs1800896 Primer G vs. Primer A genotype (size: 484 bp), IL-10 rs1800871 Primer C vs. Primer T genotype (size: 222 bp), IL-6 rs1800795 Primer G vs. Primer C genotype (size: 307 bp) for six different subjects. Odd number lanes represent wild type and even number lanes represent mutant type. Lane M = 100 bp DNA Ladder.

**Figure 2:**
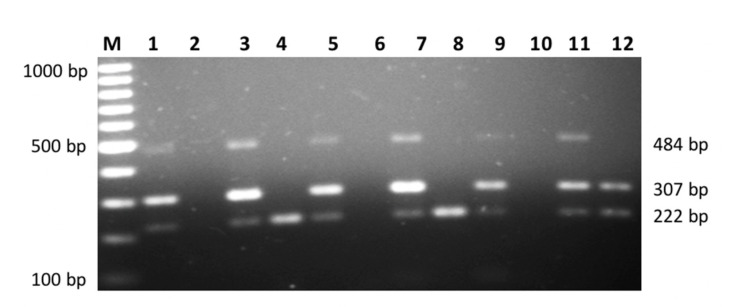
Multiplex PCR consist of TNF-α rs1800629 Primer A vs. Primer G genotype (size: 361 bp), TGF-β1 rs1800471 Primer C vs. Primer G genotype (size: 237 bp) for six different subjects. Odd number lanes represent wild type and even number lanes represent mutant type. Lane M = 100 bp DNA Ladder.

The PCR amplification was performed in 25 µL reaction volume containing a mixture of 1× PCR Buffer with KCL, 1.5–2 m Mol MgCL2, 0.1 m Mol dNTP, 0.2–0.4 m Mol forward and reverse primer, 1–2 units Taq DNA and 80–100 ng DNA. The mixtures underwent 30 cycles of the following thermocycler PCR conditions: initial denaturation at 95 °C for 3 min, denaturation at 95 °C for 30 s, annealing temperature (64 °C for 30 s for a combination of IL-10 and IL-6 primers and 69 °C for a combination of TNF-α and TGF-β1 primers), extension at 72 °C for 30 s and final extension at 72 °C for 10 min. The amplified fragments of multiplex PCR products were detected through electrophoreses of 2% agarose gel (Promega) and visualized with 1% ethidium bromide (Figure 1 and Figure 2).

**Figure 3:**
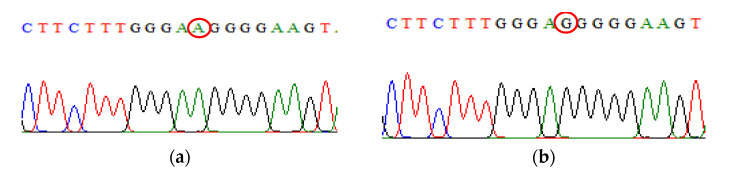
Direct DNA sequencing result for IL-10 rs1800896 polymorphism. (**a**) The chromatogram of IL-10 rs1800896 wild-type sequence (**b**) The chromatogram of IL-10 rs1800896 mutant-type sequence.

**Figure 4:**
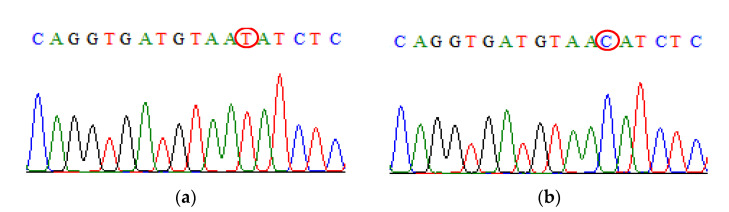
Direct DNA sequencing result for IL-10 rs1800871 polymorphism. (**a**) The chromatogram of IL-10 rs180071 wild-type sequence (**b**) The chromatogram of IL-10 rs1800871 mutant-type sequence.

**Figure 5:**
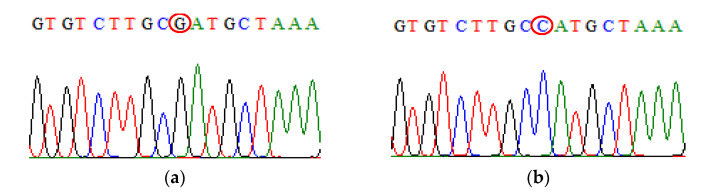
Direct DNA sequencing result for IL-6 rs1800795 polymorphism. (**a**) The chromatogram of IL-6 rs1800795 wild-type sequence (**b**) The chromatogram of IL-10 rs1800871 mutant-type sequence.

**Figure 6:**
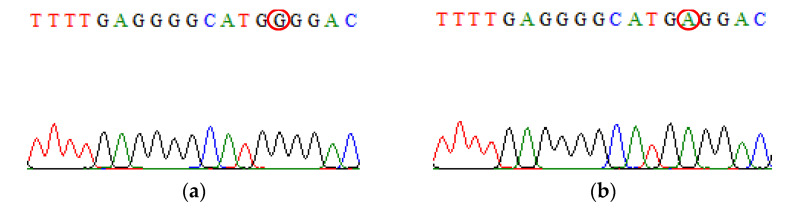
Direct DNA sequencing result for TNF-α rs1800629 polymorphism. (**a**) The chromatogram of TNF-α rs1800629 wild-type sequence (**b**) The chromatogram of TNF-α rs1800629 mutant-type sequence.

**Figure 7:**
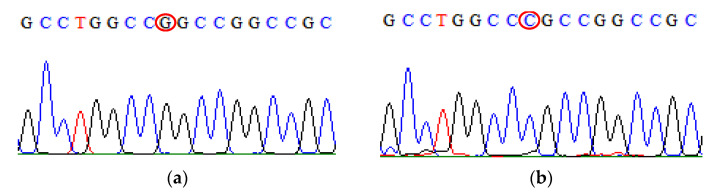
Direct DNA sequencing result for TGF-β1 rs1800471 polymorphism. (**a**) The chromatogram of TGF-β1 rs1800471 wild-type sequence (**b**) The chromatogram of TGF-β1 rs1800471 mutant-type sequence.

Upon successful PCR, some PCR products were chosen at random and sent for sequencing. The sequencing process was performed by using sequencing using the Applied Biosystems 3730 XL Genetic Analyzer (Applied Biosystems, Foster City, CA, USA), and the BigDye^®^ Terminator v3.1 cycle sequencing kit (Invitrogen, Thermo Fisher Scientific, MA, USA) was used for the sequence confirmation (Figure 3, Figure 4, Figure 5, Figure 6, Figure 7).

### 2.4. Statistical Analysis

Statistical analysis was carried out using GraphPad Prism Version 9.0. Independent-sample *t*-test and Mann–Whitney tests were used to analyze the demographic data of the subjects. The frequencies of the alleles and genotypes were calculated using the Hardy-Weinberg equation. The non-parametric chi-square test or Fischer exact test was performed to calculate the significance of the genotypes and allele frequencies among subjects. A *p* value of <0.05 was considered statistically significant.

## 3. Results

Table 2 presents the demographic and liver function test results for the 116 subjects enrolled in the study. The genotype and allele frequencies of polymorphism in IL-10 (rs1800896, rs1800871), IL-6 (rs1800795), TNF-α (rs1800629) and TGF-β1 (rs18004710) between the drug abusers with chronic HCV infection (HP) and control group (drug abusers without chronic HCV infection) are presented in Table 3.

All subjects recruited were similar in terms of demographic background. Subjects from drug abusers with chronic HCV (HP) group had significantly higher total bilirubin level compared to the control group (*p* = 0.0148). The mean AST level was abnormal for both groups as well as for GGT level for the HP group. However, the differences were not statistically significant.

The frequency of the rs1800896 AA genotype was slightly lower in the HP group (81.6%) than in the control group (95%). The rs1800896 AG and GG genotypes were slightly higher in the HP group (11.2% and 6.6%, respectively) than in the control group (5% and 0%, respectively). Overall, there were no significant differences in genotype frequencies for IL-10 rs1800896 between the HP group and the control group (*p* = 0.1600). The difference between the HP and control groups was statistically significant at the allelic level (*p* = 0.0142), with the A allele found to be higher in the control group, while the G allele was higher in the HP group.

As for IL-10 rs1800871, there were significant differences in genotype frequencies between the HP group and the control group (*p* = 0.0386). The frequency of rs1800871 TT was higher in the HP group (39.5%) than in the control group (27.5%), while for the TC genotype, the frequency was significantly lower in the HP group (40.8%) than in the control (65%). The CC genotype was also higher in the HP group (19.7%) compared to control (7.5%). However, at the allelic level, there was no significant difference between the T and C alleles in the two groups (*p* > 0.9999).

The frequency for IL-6 rs18008795 GG was slightly higher in the HP group (98.7%) than in the control (95%), whereas the GC and CC genotypes were slightly higher in the control group than in the HP group (2.5%, 2.5% vs. 1.3%, 0% respectively). However, these differences were not statistically significant (*p* = 0.4228). There was no significant difference between the G and C alleles in the two groups (*p* = 0.1196).

No significant differences were observed in the HP group compared to the control group for genotype for TNF-α rs1800629 (*p* = 0.2544). The distribution for GG, GA, and AA genotypes was almost similar in both the HP and control groups (GA = 96.1% vs. 95%, GA = 1.3% vs. 5%, and 2.6% vs. none, respectively). The distribution of G and A alleles between the two studied groups was not statistically significant (*p* = 0.1196).

There were no significant differences in genotype for TGF-β1 rs1800471 in both the HP and control groups (*p* = 0.1291). The GG genotype frequency for rs1800471 in the HP group was 88.82%, which was much lower than in the control group (97.5%). The frequency of the GC and CC genotypes in the HP group was 9.2% and 2.6%, respectively. By contrast, there was no GC genotype, and only 2.5% presented with the CC genotype in the control group. There were also no significant differences in the G and C allele distribution between the HP and control groups (*p* = 0.2282), although the frequency of the G allele was higher in the control group, while the C allele was more frequent in the HP group.

## 4. Discussion

About 60–80% of individuals infected with HCV infection are unable to spontaneously clear the virus and thus develop persistent chronic infection. As the virus itself does not cause direct liver injury, the pathophysiology of chronic HCV infection appears to be related to the local immune response. One of the crucial immune responses contributing to the pathophysiology of HCV infection is the release of cytokines. The action of cytokines can result in anti- or pro-inflammatory effects and growth stimulation or inhibition [12]. Several studies have shown that the host genetic factor plays a significant role in this immune response and the outcome of HCV infection [13].

In this study, we investigated the association of cytokine gene polymorphisms in IL-10, IL-6, TNF-α and TGF-β1 allele with susceptibility to HCV infection among Malay male drug abusers in comparison to drug abusers without HCV (control subjects). Drug abusers represent an excellent population for this study, as the prevalence of HCV infection is high with intravenous drug usage. Furthermore, as drug addictions remain one of the major health and social issues in Malaysia and worldwide, this study could provide better insights into understanding health issues among drug abusers and contribute to better management in the future. In this study, the detection of gene polymorphism was detected using the multiplex PCR method. This method is cost effective (less reagents, such as dNTPs and enzymes, are consumed), time effective, generates more data from less sample and starting materials, and, overall, increases data accuracy.

We investigated the gene polymorphism for IL-10 in its two loci, rs1800896 and rs1800871, which are both located at the promoter region. For rs1800896, we found that there were no statistical differences in frequencies for AA, AG, and GG genotypes between the group of drug abusers with chronic HCV infection and the drug abusers without HCV (control subjects). However, there were significant differences found in the allele frequency between the two studied groups, where the A allele was found to be more common than the G allele. Our findings are in contrast with previous reports of a significant association between the gene polymorphism of IL-10 rs1800896 and susceptibility to chronic HCV infection [14,15]. On the other hand, another study found no significant association between the gene polymorphism IL-10 rs1800896 and persistent HCV infection or spontaneous viral clearance [16]. A case-control study of 440 patients infected with HCV genotype 4 and 220 healthy controls in Egypt also indicated that no association was found between IL-10 rs1800896 gene polymorphisms and HCV infection [17].

As for IL-10 rs1800871, we observed significant differences in genotype distribution between drug abusers with chronic HCV infection and the control group. The rs1800871 TT genotype was found to be higher in chronic HCV infection patients compared to healthy subjects, whereas the TC genotype was higher in healthy subjects. The CC genotype was slightly higher in the HCV patient group compared to the control group. The T allele was more common than the C allele, but the differences between the two groups were not statistically significant. However, these findings are in contrast to several other studies. A study conducted on chronic HCV infection patients in a Chinese Han population did not observe any significant association between the gene polymorphism of IL-10 rs1800871 and susceptibility to HCV infection [18]. Conversely, they found that the gene polymorphism of rs1800871 played a significant role in patients’ response towards antiviral therapy. A similar finding was observed in a study on a Brazilian population, where there were no significant differences in gene polymorphism between the HCV-infected group and healthy individuals [19]. Interestingly, a study reported that the gene polymorphism of IL-10 −819 C/T was significantly associated with susceptibility to HCV infection [20]. However, the significant differences were at the allelic (C > T) level rather than genotype distribution.

Our study suggested significant associations between the gene polymorphisms of IL-10 rs1800871, IL-10 rs1800896 (at the allelic level) and susceptibility to HCV infection among drug abusers. In general, interleukin-10 plays a role as an anti-inflammatory cytokine. It is mainly produced by monocytes and lymphocytes and is encoded by the IL-10 gene located on chromosome 1q31-32 [21]. IL-10 downregulates the expression of the major histocompatibility antigens expressed by cells, which results in a reduced immune response to an antigen [22]. The single nucleotide polymorphism of the IL-10 gene in the promoter region could disrupt the transcription rate and secretion of IL-10 and affect its anti-inflammatory peripheral effects in combatting disease progression [23]. This could predispose drug abusers with IL-10 gene polymorphism to chronic HCV infection, with the A allele for rs1800896 and the TC genotype for rs1800871 seeming to be protective.

We also investigated the role of the IL-6 rs1800795 SNP in chronic HCV infection and found that GG was the most common genotype, with the G allele being the most frequent in both drug abusers with chronic HCV and the control group, and there were no significant differences. The IL-6 gene is located on human chromosome 7p21, and it is secreted by monocytes and macrophages. It can produce both pro-inflammatory and anti-inflammatory effects, and dysregulation in its synthesis plays a role in chronic inflammation and autoimmune disease [24]. However, our study could not establish an association between the IL-6 rs1800871 SNP and chronic HCV infection. The findings are in contrast with a recent meta-analysis study that suggested a strong association between the gene polymorphism of IL-6 rs1800795 carrying the G allele and susceptibility to liver disease [25]. This observation is supported by studies that established an association between the gene polymorphism of IL-6 rs1800871 and the outcome of HCV infection [16,26]. Furthermore, a study involving an Egyptian population found a significant increase in G alleles in all IL-6 polymorphisms (−174 G/C), (−597G/A), and (−572 G/C), thus suggesting that a predisposition to chronic HCV infection is associated with these genes in their population [27].

TNF-α is a pro-inflammatory cytokine released by activated macrophages in response to bacterial endotoxins. It is encoded by the TNF-α gene located at human chromosomes 6p21.3 9 [28]. The polymorphism in the promoter region could affect the transcription rate and the secretion of TNF-α [29]. However, in this study there were no significant differences in genotype and alleles for TNF-α (rs1800629) between chronic HCV infection and the control group. One study involving an Egyptian population also found that none of the TNF-α −1031 T/C, −863 C/A, −857 C/T, and −308 G/A polymorphisms were associated with HCV infection [30]. By contrast, some studies have established the association of the gene polymorphism of the TNF-α rs1800629 with susceptibility to HCV infection. TNF-α A/G and A/A genotypes have been significantly associated with susceptibility to hepatitis C infection [17], and a significant association between TNF-α −308 G/G genotype and HCV infection was seen in patients compared with healthy individuals [31]. TNF-α −308 G/A polymorphism was also reported to have a link to the pathogenesis and advancement of chronic hepatitis C [32,33].

Regarding TGF-β1 rs1800471, we found no significant differences in genotype and allele distribution between the chronic HCV infection group and the control group. The distribution of the GG genotype and G allele was lower but not statistically significant in the drug abusers with chronic HCV infection compared to the control group. These findings are different with some past studies that indicated an association of the gene polymorphism of the TGF-β1 genotype with the outcome of HCV infection [14]. By contrast, several studies that explored the role of TGF-β1 rs1800471 (the codon 25G) polymorphism in chronic HCV infection in Asian, Caucasian, and Brazilian populations found no significant association between the codon 25G/C polymorphism and chronic HCV infection in all subgroup analyses [16,34,35]. The human TGF-β1 gene located on chromosome 19q13 is mainly expressed by regulatory T cells [36]. TGF-β1 plays multifunctional roles, encoding proteins responsible for differentiation and apoptosis, and it exerts strong anti-inflammatory effects [37]. The findings of our study suggest that the TGF-β1 rs1800471 SNP with mutation of G allele to C allele does not significantly predispose the drug abuser group to the development of chronic HCV infection.

Some of our findings confirmed the results from previous studies, whereas others were in conflict with previous observations. These contradictions in gene polymorphism studies could be due to various factors, such as sample size differences, subject’s selection, genetic heterogeneity in different ethnicities, and different gene–gene or gene–environment interaction [20]. On the other hand, this study has several notable limitations. The number of subjects recruited was small and the frequencies for some genotypes of SNPs were low. These limitations may restrict the statistical power; thus, our results should be interpreted with caution. The study can be improved in the future by overcoming these limitations, as well as extending the study to the role of gene polymorphisms on treatment response.

## 5. Conclusions

A simple and rapid method of multiplex PCR was used for the simultaneous detection of IL-10 rs1800896 and rs1800871, IL-6 rs1800795, TNF-α rs1800629, and TGF-β1 rs18004710 gene polymorphisms. The findings of this study suggest that the gene polymorphisms of IL-10 rs1800871 and IL-10 rs1800896 (at the allelic level) were associated with susceptibility to the development of chronic HCV infection among drug abusers. Further investigation is warranted to establish a true clinical significance for these findings.

## Figures and Tables

**Table 1:** Primers sequence for genes of interest.

SNP	Primer	Primer Sequences (5′ → 3′)	Size (bp)
IL-10 rs1800896	Primer A Primer G	Forward: ACTACTAAGGCTTCTTTGGGAA Forward: CTACTAAGGCTTCTTTGGGAG Reverse: TACCCTTGTACAGGTGATGTAAT	484 484
IL-10 rs1800871	Primer T Primer C	Forward: TACCCTTGTACAGGTGATGTAAT Forward: TACCCTTGTACAGGTGATGTAAC Reverse: TACCCTTGTACAGGTGATGTAAT	222 222
IL-6 rs1800795	Primer G Primer C	Forward: CCCCCCTAGTTGTGTCTTGCG Forward: CCCCCTAGTTGTGTCTTGCC Reverse: CAGTTCCAGGGCTAAGGATTTC	307 307
TNF-α rs1800629	Primer G Primer A	Forward: GCAATAGGTTTTGAGGGGCATGG Forward: GCAATAGGTTTTGAGGGCATGA Reverse: TGCTGTCCTTGCTGAGGGAGC	361 361
TGF-β1 rs1800471	Primer G Primer C	Forward: TACTGGTGCTGACGCCTGGCCG Forward: TACTGGTGCTGACGCCTGGCCC Reverse: GCTCCGGTTCTGCACTCTCCC	237 237

**Table 2:** Demography and serum liver function test (LFT) for all subjects.

Characteristics	Drug Abusers with Positive Chronic HCV (HP Group) (*n* = 76) Mean (SD)	Drug Abusers without HCV (Control Group) (*n* = 40) Mean (SD)	Statistics *p*-Value
Age (years) ^a^	42.50 (5.24) ^c^	43.00 (6.90) ^c^	0.2809
Height (m) ^b^	163.8 (5.37)	164.5 (7.58)	0.5789
Weight (kg) ^b^	60.03 (8.70)	60.88 (10.12)	0.6396
BMI (kg/m^2^) ^b^	22.40 (3.31)	24.03 (3.00)	0.5578
Brachial systolic BP (mm/Hg)	121.6 (16.04)	121.7 (14.05)	0.9676
Brachial diastolic BP (mm/Hg)	79.83 (9.74)	78.75 (8.69)	0.5576
Total protein (g/L)	80.51 (8.53)	79.78 (8.95)	0.6959
Albumin (g/L)	39.40 (3.90)	40.92 (3.30)	0.0576
Total bilirubin (umol/L)	9.48 (8.18)	5.984 (2.50)	* 0.0148
Alanine aminotransferase, ALT (U/L)	35.89 (29.74)	42.33 (42.29)	0.3957
Aspartate transaminase, AST (U/L)	51.62 (32.25)	49.00 (35.88)	0.7181
Alkaline phosphatase, ALP (U/L)	110.2 (46.6)	95.53 (39.21)	0.1224
Gamma-glutamyl transpeptidase, GGT (U/L)	97.38 (159.5)	62.36 (61.29)	0.2123

^a^ Mann-Whitney test. ^b^ Independent-samples *t*-test. ^c^ Median (IQR). * Significant difference between control and drug abusers with chronic HCV groups with *p* < 0.05.

**Table 3:** Allelic and genotypic frequencies of IL-10 (rs1800896 and rs1800871), IL-6 (rs1800795), TNF-α (rs1800629), and TGF-β1 (rs1800471) polymorphism in drug abusers with chronic HCV and control subjects.

SNP	Genotype and Alleles	Drug Abusers with Chronic HCV (HP Group) (*n* = 76)	Drug Abusers without HCV (Control Group) (*n* = 40)	Statistics ^a^ *p*-Value
IL-10 rs1800896	AA	62 (81.6%)	38 (95%)	0.16
	AG	9 (11.2%)	2 (5%)	
	GG	5 (6.6%)	0 (%)	
	A	133 (87.5%)	78 (97.5%)	* 0.0142
	G	19 (12.5%)	2 (2.5%)	
IL-10 rs1800871	TT	30 (39.5%)	11 (27.5%)	* 0.0386
	TC	31 (40.8%)	26 (65%)	
	CC	15 (19.7%)	3 (7.5%)	
	T	91 (59.9%)	48 (60%)	>0.9999
	C	61 (40.1%)	32 (40%)	
IL-6 rs1800795	GG	75 (98.7%)	38 (95%)	0.4228
	GC	1 (1.3%)	1 (2.5%)	
	CC	0	1 (2.5%)	
	G	151 (99.3%)	77 (96.25%)	0.1196
	C	1 (0.7%)	3 (3.75%)	
TNF-α rs1800629	GG	73 (96.1%)	38 (95%)	0.2544
	GA	1 (1.3%)	2 (5%)	
	AA	2 (2.6%)	0	
	G	147 (96.7%)	78 (97.5%)	>0.9999
	A	5 (3.3%)	2 (2.5%)	
TGF-β 1rs1800471	GG	67 (88.2%)	39 (97.5%)	0.1291
	GC	7 (9.2%)	0	
	CC	2 (2.6%)	1 (2.5%)	
	G	141 (92.8%)	78 (97.5%)	0.2282
	C	11 (7.2%)	2 (2.5%)	

^a^ Chi-squared test/Fischer exact test where appropriate. * Significant difference between control and drug abusers with chronic HCV groups with *p* < 0.05.

## Data Availability

The data used and analyzed in this study are available from the corresponding author on request. This will require an ethical permit.
